# Validation of the Spanish version of the borderline symptom list, short form (BSL-23)

**DOI:** 10.1186/1471-244X-13-139

**Published:** 2013-05-14

**Authors:** Joaquim Soler, Daniel Vega, Albert Feliu-Soler, Joan Trujols, Ángel Soto, Matilde Elices, Cristina Ortiz, Víctor Pérez, Martin Bohus, Juan Carlos Pascual

**Affiliations:** 1 Servicio de Psiquiatría, Hospital de la Santa Creu i Sant Pau, Universitat Autònoma de Barcelona, Avda Sant Antoni Mª Claret 167, Barcelona 08025, Spain; 2 Centro de Investigación Biomédica en Red de Salud Mental (CIBERSAM), Madrid, Spain; 3 Servei de Psiquiatria i Salut Mental, Hospital de Igualada (Consorci Sanitari de l’Anoia), Igualada, Spain; 4 Unitat de Psicologia Mèdica, Departament de Psiquiatria i Medicina Legal & Institut de Neurociències, Universitat Autònoma de Barcelona, Barcelona, Spain; 5 Department of Psychosomatics and Psychotherapy, Central Institute of Mental Health, Manheim, Germany

**Keywords:** Borderline personality disorder, Borderline symptom list, Instrumental study, BPD severity

## Abstract

**Background:**

The Borderline Symptom List-23 (BSL-23) is a reliable and valid self-report instrument for assessing Borderline Personality Disorder (BPD) severity. The psychometric properties of the original version have proven to be adequate. The aim of the present study was to validate the Spanish language version of the BSL-23.

**Methods:**

The BSL-23 was administered to 240 subjects with BPD diagnosis. Factor structure, reliability, test-retest stability, convergent validity, and sensitivity to change were analyzed.

**Results:**

The Spanish version of the BSL-23 replicates the one-factor structure of the original version. The scale has high reliability (Cronbach’s alpha=.949), as well as good test-retest stability, which was checked in a subsample (*n*=74; *r*=.734; *p*<.01). The Spanish BSL-23 shows moderate to high correlations with depressive symptomatology, state and trait anxiety, hostility and impulsivity scores and BPD measures. The Spanish BSL-23 is able to discriminate among different levels of BPD severity and shows satisfactory sensitivity to change after treatment, which was verified by assessing change before and after 12 group sessions of Dialectical Behavioral Therapy in a subgroup of 31 subjects.

**Conclusions:**

Similar to the original BSL-23, the Spanish BSL-23 is a reliable and valid instrument for assessing BPD severity and sensitivity to change.

## Background

Borderline Personality Disorder (BPD) is a severe psychiatric condition affecting from 1 to 5.9% of the general population [1,2]. However, in clinical populations, BPD is prevalent in up to 25% of inpatients [3]. According to the DSM-IV criteria [4], BPD is characterized by a pervasive pattern of instability in interpersonal relationships, identity, impulsivity, and affect. BPD is associated with high rates of suicide [5], comorbid Axis I and II mental disorders [6-8], severe functional impairment [9], and high costs for psychiatric services [9,10]. BPD diagnosis is generally based on (1) generic Axis II diagnostic interviews that include all DSM-IV Axis II diagnoses such as the SCID-II [11] or (2) BPD-specific structured interviews such as the Diagnostic Interview for Borderlines-Revised (DIB-R) [12,13], which improve the specificity and accuracy of the diagnosis.

Due to the wide range of BPD symptoms, studies of this disorder have used multiple symptomatology scales designed to evaluate Axis I disorders. The use of several different scales gives an indirect assessment of BPD symptoms. However, more recently, several BPD-specific scales based on DSM-IV criteria have been developed to increase accuracy of the diagnosis and decrease the time needed to perform the assessment. Clinician-administered scales include the BPD Severity Index IV (BPDSI-IV) [14], the Zanarini Rating Scale for Borderline Personality Disorder (ZAN-BPD) [15], and the Clinical Global Impression scale for BPD patients (CGI-BPD) [16]. Differences between these scales mainly involve administration time, time-frame considered, and the number of items used to assess each criterion. Despite the good psychometric properties of these clinician-administered instruments, they all have certain disadvantages, such as a long administration time or a need for clinical expertise to perform the assessments.

At the moment, only three self-reported scales are available to assess BPD severity. The Borderline Evaluation of Severity Over Time (BEST) [17], a 15-item instrument composed of 3 subscales to assess the thoughts, emotions, and behaviours typical of BPD subjects and to measure severity and changes in BPD. The second instrument is the Borderline Symptom List (BSL-95), created by Bohus et al. [18] based on DSM-IV criteria and the DIB-R, including the collaboration of clinical experts and patients. The BSL-95 contains 95 items to evaluate the subjective complaints common among BPD subjects. Each item is quantitatively assessed on a 5-point Likert scale that ranges from 0 (not at all) to 4 (very strong). The BSL-95 has strong psychometric properties, but its length makes it impractical in some settings. For this reason, the original version (BSL-95) was later reduced to create the Borderline Symptom List-23 (BSL-23) [19]. The BSL-23 includes those items from the original version that had shown the highest levels of sensitivity to change and that discriminate best between BPD and other disorders. In addition, because the new version contains only 23 items, the administration time is considerably shortened. The psychometric properties of the BSL-23 were assessed in 5 different BPD patient samples. A total of 659 subjects were evaluated and correlation between the BSL-95 and the BSL-23 was high. The principal component analysis suggested a structure of one dominant factor. Findings showed a high internal consistency, high test-retest reliability, and good sensitivity to change after Dialectical Behavioral Therapy (DBT) treatment. In short, the authors considered that the BSL-23 was a reliable and brief self-reported instrument for assessing BPD severity as well as sensitivity to change.

Several instruments have been created to assess BPD diagnosis and severity in the Spanish population [13,16]. However, no validated self-report measures are yet available to specifically assess BPD severity. For this reason, the aim of the present study was to validate the Spanish language version of the BSL-23 in a sample of subjects with BPD diagnosis. The psychometric properties of Spanish version of the BSL-23 and its sensitivity to change due to therapeutic intervention were tested.

## Method

### Participants

The total sample consisted of 240 subjects recruited from mental health settings in Spanish public institutions. Sample size was considered appropriate taking into account psychometric recommendations that suggest 5 to 10 individuals per item [20]. Inclusion criteria consisted of BPD diagnosis according to DSM-IV criteria as assessed by a structured interview (DIB-R) [13] and age between 18 and 45 years. Exclusion criteria were as follows: comorbidity with bipolar disorder, schizophrenia, current major depressive disorder, substance dependence, and severe difficulties in reading comprehension. All participants had to be native Spanish speakers.

This study was approved by the Clinical Research Ethics Committee at the Hospital de la Santa Creu i Sant Pau and carried out in accordance with the Declaration of Helsinki. Participants were given a detailed description of the study and gave their written informed consent.

### Instruments

### Diagnosis interview

–Revised Diagnostic Interview for Borderlines (DIB-R) [13]. The DIB-R is a structured interview for assessing BPD diagnosis criteria in four areas: impulsive behavior, cognitive area, affective area and interpersonal relationships. The assessment focuses on the prior two years and the scale ranges from 0 to 10, with a cut-off level set at 6 for a diagnosis of BPD [13].

### Scales

– Borderline Symptom List – 23 (BSL-23) [19]. The BSL-23 is a self-rated scale that assesses BPD symptomatology. The original version is composed of a one-factor structure and has shown a high internal consistency as evidenced by a Cronbach’s alpha of 0.93. It has also shown good reliability for BPD diagnosis as well as a satisfactory sensitivity to change. Administration of the BSL-23 takes an average of 3 to 4 minutes.

– Barrat Impulsivity Scale (BIS-11) [21]. The BIS-11 is a 30-item self-reported scale that contains 3 subscales: motor impulsivity, cognitive impulsivity, and lack of planning. The global impulsivity score ranges between 0 and 120.

– Beck Depression Inventory (BDI-II) [22]. This self-report instrument consists of 21 items to evaluate depressive symptomatology.

– State Trait Anxiety Inventory (STAI) [23]. STAI is a 40-item questionnaire with two subscales (20 items each), one to assess state anxiety (STAI-S) and the other to evaluate trait anxiety (STAI-T). Total scores range from 0 to 60, and there is no clinical cut-off in the Spanish version.

– Buss-Durkee Hostility Inventory (BDHI) [24]. The BDHI is a self-reported true/false questionnaire with 75 items, 7 subscales (Assault, Indirect Hostility, Irritability, Negativism, Resentment, Suspicion, and Verbal Hostility) and a global hostility score.

– Clinical Global Impression for Borderlines (CGI-BPD) [16]. The CGI-BPD assesses the severity of BPD symptoms as well as sensitivity to therapeutic change. This clinician-administered scale measures the severity of nine items using a 7-point Likert scale. The items are based on DSM-IV diagnosis criteria for BPD. In our study, the CGI-BPD was only administered to a sub-sample of patients to assess sensitivity to change.

### Procedure

Participants were enrolled in the study over a two-year period according to the predetermined inclusion and exclusion criteria. Subjects received information about the study aims and instructions on how to complete the tests. Participation in the study was voluntary and no financial retribution was given.

The BSL-23 was translated from English to Spanish by three native Spanish speakers with clinical expertise and in accordance with the author’s supervision. An independent native English-speaking translator with expertise in translation of bio-medical texts translated this Spanish version back into English. The authors of the original BSL-23 version approved the final Spanish version.

To analyze the convergent validity of the Spanish BSL-23, the following scales were used: BIS-11, BDI, STAI-S, STAI-T, BDHI and CGI-BPD (this latter available only from 31 individuals). Correlation between BSL-23 and DIB-R was also performed in order to ascertain the convergence with a diagnostic instrument for BPD. To study test-retest reliability of the Spanish BSL-23, a sub-sample of 74 participants were asked to complete the instrument again after one week. To examine sensitivity to change following treatment, 31 individuals completed the BSL-23 before and after 3 months intervention based on DBT in a group format (1 session per week). The DBT group therapy consists of a skills training 2 hours per week. These skills are divided into four modules: Interpersonal Effectiveness, Emotion Regulation, Distress Tolerance and Mindfulness. This sub-sample also completed the CGI-BPD before and after treatment.

In order to test if BSL-23 was able to differentiate among different levels of BPD severity, the following procedure was used. A stepwise backward linear regression analysis was performed including BDI for affective symptomatology, BIS-11 subscales for impulsivity, and BDHI subscales for hostility and aggression as independent variables to predict CGI-BPD scores as the dependent variable. BDI total score, BIS-11 non-planning impulsivity and all BDHI subscales except “Distrust” significantly predicted CGI-BPD scores (R^2^=.73; p=.03). The unstandardized values from the regression model were then divided into quartiles, which served to finally classify participants in four categories of BPD severity. This variable was introduced as between-group factor in a one-way ANOVA with BSL-23 scores as the dependent variable.

### Data analysis

Data analysis was carried out using SPSS 18.0 statistical software for Windows. Descriptive statistics were used to describe the demographic and clinical characteristics of the sample. An exploratory factorial analysis (EFA) of principal components with a Varimax rotation was performed to examine the factorial structure of the scale. It was used Confirmatory Factor Analysis (CFA) to explore the goodness of fit of the original one-factor structure of the BSL. EQS software for Windows version 6.1 [25] was used to conduct the CFA. The maximum likelihood with robust correction method was used to adjust for distributional problems in the data set. Although a model with a non-significant chi-square estimate is generally considered a model with good fit, Hu and Bentler [26] recommended combinational rules to evaluate model fit. The following criteria were used to indicate the fit of the CFA models to the data: CFI (Comparative Fit Index) and GFI (Goodness of Fit Index) >.90 and RMSEA (Root Mean Square Error of Approximation) <.08. Values for CFI and GFI ranged from 0 to 1. These fit statistics and the chi-square were selected because previous research has demonstrated their performance and stability [26,27].

To test internal consistency, global Cronbach’s alpha was estimated and the split-half method was also applied. In addition, Cronbach’s alpha was estimated with each of the 23 items removed one at a time from the scale. Test-retest reliability and convergent validity were evaluated by correlation analysis. In order to assess the BSL-23 sensitivity to clinical change, we analyzed post- minus pre- treatment scores using a t-test comparison. Additionally, we compared this change value with CGI-BPD results through correlational analysis.

## Results

### Socio-demographic and clinical characteristics of the sample

As shown in Table 1, more than half of the sample were women (57.5%), with a mean age of 32.4 years old (*SD*=8). A slight majority were single (52.8%) and most had completed at least secondary schooling. Most subjects (80%) were not working or studying at the time of the study. BPD symptomatology was moderate to severe, and the mean DIB-R score was 7.4 (*SD*=1.6).

**Table 1:** Socio-demographic and clinical characteristics of the sample

	**BPD ( *n *=240)**
Age (*M, SD*)	32.4 (8)
DIB- R (*M, SD*)	7.4 (1.6)
Gender – Woman (%)	57.5
Marital status (%)	
Single	52.8
Married	32.3
Divorced	14.5
Widow	.4
Education (%)	
No education	3.8
Primary school	34.6
Secondary education	44.2
University	15.4
Current activity (%)	
Employed	16.7
Student	3.3
unemployed	80%

Note. BPD=Borderline Personality Disorder; DIB-R=Diagnostic Interview for Borderlines Revised.

### Factor structure

**Figure 1:**
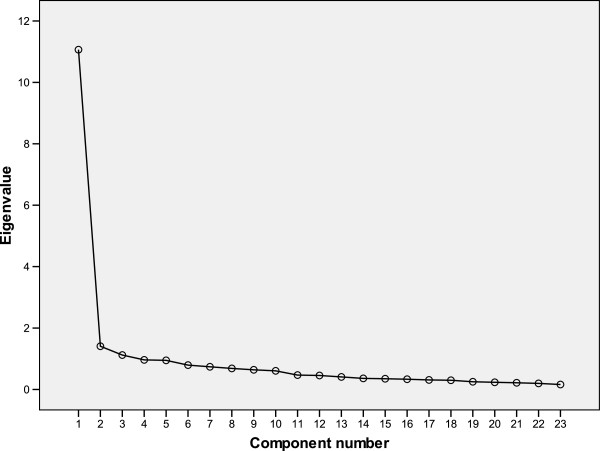
Scree plot of the BSL-23 Spanish version.

The Kaiser-Meyer-Olkin (KMO) measure yielded a value of .952 and Bartlett’s Test of Sphericity was significant (*p*<.001). An initial exploratory component analysis with a Varimax rotation showed a three factor structure with Eigen values greater than 1 (11.065, 1.407 and 1.120), accounting for 59.1% of total variance. The scree plot, however, suggested a unifactorial solution (Figure 1). A posterior EFA was performed by fixing one factor. The unifactorial solution explained 48.11% of total variance. Most of the items (22 out of 23) showed total factorial loadings superior to .40, except for item 23. Table 2 shows the factorial loadings of each item.

**Table 2:** Factor Structure of the BSL-23 Spanish version

**BSL Items**		**Factor 1**
BSL-1	Me resultaba difícil concentrarme	.702
BSL-2	Me sentí indefenso	.737
BSL-3	Estuve ausente e incapaz de recordar que estaba haciendo en realidad	.606
BSL-4	Sentí asco	.569
BSL-5	Pensé en hacerme daño	.767
BSL-6	Desconfié de los demás	.586
BSL-7	No creía que tuviera derecho a vivir	.775
BSL-8	Me sentía solo	.749
BSL-9	Sentí una tensión interna estresante	.800
BSL-10	Sentí mucho miedo de imágenes que me vinieron a la cabeza	.655
BSL-11	Me odié a mí mismo	.825
BSL-12	Quise castigarme	.603
BSL-13	Sufrí de vergüenza	.640
BSL-14	Mi humor oscilaba rápidamente entre la ansiedad, la rabia y la depresión	.831
BSL-15	Sufrí al oír voces y ruidos procedentes de dentro o fuera de mi cabeza	.521
BSL-16	Las críticas tuvieron un efecto demoledor en mí	.711
BSL-17	Me sentí vulnerable	.752
BSL-18	La idea de morirme me causó una cierta fascinación	.782
BSL-19	Nada parecía tener sentido para mí	.839
BSL-20	Tuve miedo de perder el control	.797
BSL-21	Me di asco a mí mismo	.600
BSL-22	Tuve la sensación de salir de mí mismo	.490
BSL-23	Sentí que no valía nada	.373

The one-factor model in the CFA showed good fit indices _sb_χ^2^= 574.7904 (p<.001), (CFI= .905, GFI=.767, SRMR=.053, RMSEA= .079 [.071-.087]). According to the fit indices, the one-factor model represents correctly the observed data.

### Reliability

The Cronbach’s alpha coefficient was .936 and the split-half method yielded a correlation of *r*=.92, indicating that the scale has a high overall internal consistency. In the item-by-item reliability analysis, Cronbach's alpha coefficient showed a slight improvement (from .936 to .949) in its value when item 23 was not included.

Analysis of test-retest stability within one week was performed in a subsample of 74 subjects, with satisfactory results (*r*=.734; *p*<.01).

### Convergent validity

As shown in Table 3*,* positive moderate to high correlations were observed between the scores on the BSL-23 and depression symptoms measured by the BDI (*r*=.787), state-anxiety measured by the STAI-S (*r*=.705), trait-anxiety by means of STAI-T (*r*=.746), hostility measured by the BDHI (*r*=.421) and impulsivity measured by the BIS (r=.376). All correlations were highly significant at p< .001. Correlation analyses between BSL-23 and DIB-R and CGI-BPD showed that this latter was strongly associated with BSL-23, while DIB-R was moderately related (see Table 3).

**Table 3:** Correlations between Spanish version of BSL-23 scores and other scales

	**BSL-23**
Beck Depression Inventory (BDI)	.787**
State Trait Anxiety Inventory – S (STAI-S)	.705**
State Trait Anxiety Inventory – T (STAI-T)	.746**
Buss-Durkee Hostility Inventory (BDHI) Total Score	.421**
BDHI – Fisical Hostility	.200**
BDHI – Verbal Hostility	.244**
BDHI- Indirect Hostility	.373**
BDHI - Irritability	.396**
BDHI - Resentment	.412**
BDHI - Distrust	.333**
BDHI - Negativism	.180*
BDHI - Guilt	.301**
Barrat Impulsivness Scale (BIS) – Total Score	.376**
BIS – Attentional	.144*
BIS - Motor	.424**
BIS - Nonplanning	.276**
CGI-BPD	.889**
DIB-R	.407**

*p<.05, **p<.001.

### Sensitivity to change

To establish the scale’s ability to detect improvement in BPD symptomatology, the delta scores (difference between the first and second administration scores) obtained in BSL-23 were correlated with the improvement observed in the CGI-BPD (measured before and after a 3-month DBT skills training treatment) in a sub sample of 31 patients with BPD. The mean pre- and post-treatment scores for the BSL-23 were, respectively, 2.21 (*SD*=.96), and 1.83 (*SD*=.96; *p*= .01). For the CGI-BPD, the mean pre- and post-treatment scores were 4.80 (*SD*=1.04) and 4.16 (*SD*= .96.; *p*= .002), respectively. The mean change was obtained for both the BSL-23 and the CGI-BPD (including overall score plus all subscales) in order to compare pre- and post-treatment scores. The correlation between the mean change in BSL-23 and in the CGI-BPD global score was significant (*r*=.79). Other significant correlations were identified for the following subscales: Abandonment (*r*=.636), Unstable Relationships (*r*=.719), Impulsivity (*r*=.675), Suicide(*r*=.733), Affective Instability (*r*=.836), Anger (*r*=.810) and Paranoid Ideation (*r*=.503). All reported correlations were significant at *p*<.01.

### Discrimination among BPD severity levels

BSL-23 was able to discriminate among different levels of BPD severity [F(3,179)=51.18; p<.001]. Table 4 displays Bonferroni’s posthoc analyses regarding BSL-23 scores among groups. As it can be observed BSL-23 differentiated all groups of severity.

**Table 4:** BSL mean scores’ distribution by BPD severity levels as measured by the categorical CGI-BPD unstandardized scores

	**1st quartile (n=46)**	**2nd quartile (n=46)**	**3rd quartile (n=46)**	**4th quartile (n=45)**	**Bonferroni posthoc comparisons**
BSL-23 scores	.55 [.61]	1.03 [.81]	1.78 [.81]	2.44 [.89]	(1st )<(2nd)*,
					(1st )< (3rd)***, (1st )< (4th)***,
					(2nd)<(3rd )***, (2nd)< (4th)***,
					(3rd)<(4th)**

*p<.05; **p<.01; ***p<.001.

Mean and SD (between brackets) are represented. The following quartile values represent each severity level according to predicted CGI-BPD scores: 1st quartile: 3.59, 2nd quartile: 4.27; 3rd quartile: 4.9; 4th quartile: 6.63. BPD: Borderline Personality Disorder,.

## Discussion

Although BPD is the most commonly studied personality disorder in clinical trials, only a very limited number of self-reported questionnaires are available to specifically assess BPD severity and sensitivity to change following therapeutic interventions. Moreover, none of the existing instruments had been adapted for Spanish speakers, until now. The original BSL-95 was shortened to the BSL-23 to reduce assessment time and to target sensitivity to change. In the current study, the Spanish BSL-23 has shown good psychometric properties similar to the original version.

The principal component analysis of the current instrument presents a unidimensional factor structure identical to the original version. The percentage of variance (48.11%) explained by this single factor is even better than the one obtained in the original validation study [18]. In our study, this unidimensional model was also confirmed by a CFA. The findings show a high internal consistency of the Spanish short version of BSL as indicated by the high Cronbach’s alpha and Spearman-Brown coefficient. Although item 23 shows less psychometric robustness compared to the rest on factorial and reliability analyses, their psychometric indexes range from moderate to acceptable values. Moreover, the reliability of the Spanish BSL-23 is similar to that of the English versions (BSL-23 and BSL-95), both of which had a Cronbach’s alpha of .97.

The one-week temporal reliability of the scale was high (*r*=.73) and comparable to the scores obtained in the original BSL-23 (*r*=.82) and BSL-95 (*r*=.84) studies [18,19].

Given the wide spectrum of symptoms for BPD, we expected to observe positive correlations between the Spanish version of BSL-23 and other self-reported scales designed to assess depression and anxiety symptoms, hostility, and impulsivity. BSL-23 scores showed a positive correlation with BDI, BDHI, BIS and STAI-S and STAI-T and this correlation ranged from low/moderate –BDHI and BSI (*r*=.144 to *r*=.424; including subscales)– to high: BDI and STAI subscales (*r*=.705 to *r*=.787). The original BSL-23 study showed a strong correlation between scores on affective symptomatology and BSL-23 [19]; in our study, we also found a high correlation between BSL-23 and BDI scores. This association between depressive symptomatology and BPD scales has also been reported in other self-reported BPD scales [17]. However, unlike the original study by Bohus et al. [19], we found high and significant correlations between anxiety symptoms and BSL-23. Although measures of hostility and impulsivity were not used to study convergent validity in previous BSL validations, we found moderate and positive correlations between Spanish BSL-23 and BDHI (hostility) and BIS (impulsivity) scores. The correlations seen in our study were similar to those previously described by Bohus et al. [18]. As expected, correlations between BSL-23 and other specific BPD instruments i.e. DIB-R and CGI-BPD indicating good and excellent convergent validity, respectively. The slight discrepancy of convergent validity with regard these two instruments may be due to the fact that BSL-23 and CGI-BPD assess the same temporal frame (one week), while the DIB-R recalls information for the last two years. These results support the convergent validity of the Spanish version of the questionnaire and the capacity of the BSL-23 to differentiate different levels of severity is a strong attribute of the scale to be used either in clinical settings and research.

The instrument also showed a good capacity to detect changes produced by therapeutic BPD interventions. To assess sensitivity to changes, we carried out a 3-month DBT group therapy program whose effectiveness in BPD patients had been proven in a previous study [28]. This therapeutic intervention, based on self-regulatory skills acquisition, was nearly identical to the program used in the original BSL-23 [19]. In our study, BSL-23 scores were compared to the CGI-BPD [16], an instrument also developed to assess changes in BPD symptoms after treatment, and we found a strong positive correlation between both instruments. BSL-23 also correlate positively (*r≥*.50) with improvements observed in the CGI-BPD subscales for Abandonment, Unstable Relationships, Impulsivity, Suicide, Affective Instability, Anger Paranoid Ideation.

Finally, a limitation of our study is the absence of a non-BPD clinical group comparison as had been done in the original English version with five different psychiatric samples [19].

## Conclusions

To conclude, the Spanish BSL-23 is a reliable instrument for assessing and discriminating BPD severity and clinical outcomes after a psychotherapeutic intervention. Moreover, administration-time is brief and it is suitable for use in both research and clinical settings.

## Competing interests

The authors declare that they have no competing interests.

## Authors’ contributions

JS conceived the study aims and design, and developed the study in discussions with JT, JP, AF, CO, ME, DV, AS and MB. JS performed the analysis and drafted the initial manuscript. All authors contributed to interpretation of results, revised and commented on the manuscript for important intellectual content. All authors read and approved the final manuscript.

## Pre-publication history

The pre-publication history for this paper can be accessed here:

http://www.biomedcentral.com/1471-244X/13/139/prepub
