# Erratum to: Validation of the Spanish version of the borderline symptom list, short form (BSL-23)

**DOI:** 10.1186/s12888-016-1144-7

**Published:** 2016-12-01

**Authors:** Joaquim Soler, Daniel Vega, Albert Feliu-Soler, Joan Trujols, Ángel Soto, Matilde Elices, Cristina Ortiz, Víctor Pérez, Martin Bohus, Juan Carlos Pascual

**Affiliations:** 1 Servicio de Psiquiatría, Hospital de la Santa Creu i Sant Pau, Universitat Autònoma de Barcelona, Avda Sant Antoni Mª Claret 167, Barcelona, 08025 Spain; 2 Centro de Investigación Biomédica en Red de Salud Mental(CIBERSAM), Madrid, Spain; 3 Servei de Psiquiatria i Salut Mental, Hospital de Igualada (Consorci Sanitari de l’Anoia), Igualada, Spain; 4 Unitat de Psicologia Mèdica, Departament de Psiquiatria i Medicina Legal & Institut de Neurociències, Universitat Autònoma de Barcelona, Barcelona, Spain; 5 Department of Psychosomatics and Psychotherapy, Central Institute of Mental Health, Manheim, Germany

## Erratum

The original article [1] is missing some funding information from the Acknowledgements section. The authors would like to state the following instead:

This study was supported by Centro de Investigación Biomédica en Red de Salud Mental (CIBERSAM) and by a grant from Instituto de Salud Carlos III (PI10/00253), co-financed by the European Regional Development Fund (ERDF).
